# Scale-Invariant Multidirectional License Plate Detection with the Network Combining Indirect and Direct Branches

**DOI:** 10.3390/s21041074

**Published:** 2021-02-04

**Authors:** Song-Lu Chen, Qi Liu, Jia-Wei Ma, Chun Yang

**Affiliations:** 1 School of Computer and Communication Engineering, University of Science and Technology Beijing, Beijing 100083, China; chenslvs7@gmail.com (S.-L.C.); qiliu@xs.ustb.edu.cn (Q.L.); mjw20151001@gmail.com (J.-W.M.); 2 USTB-EEasyTech Joint Lab of Artificial Intelligence, University of Science and Technology Beijing, Beijing 100083, China

**Keywords:** license plate detection, multiscale, multidirectional, indirect branch, direct branch, end-to-end

## Abstract

As the license plate is multiscale and multidirectional in the natural scene image, its detection is challenging in many applications. In this work, a novel network that combines indirect and direct branches is proposed for license plate detection in the wild. The indirect detection branch performs small-sized vehicle plate detection with high precision in a coarse-to-fine scheme using vehicle–plate relationships. The direct detection branch detects the license plate directly in the input image, reducing false negatives in the indirect detection branch due to the miss of vehicles’ detection. We propose a universal multidirectional license plate refinement method by localizing the four corners of the license plate. Finally, we construct an end-to-end trainable network for license plate detection by combining these two branches via post-processing operations. The network can effectively detect the small-sized license plate and localize the multidirectional license plate in real applications. To our knowledge, the proposed method is the first one that combines indirect and direct methods into an end-to-end network for license plate detection. Extensive experiments verify that our method outperforms the indirect methods and direct methods significantly.

## 1. Introduction

**Figure 1:**
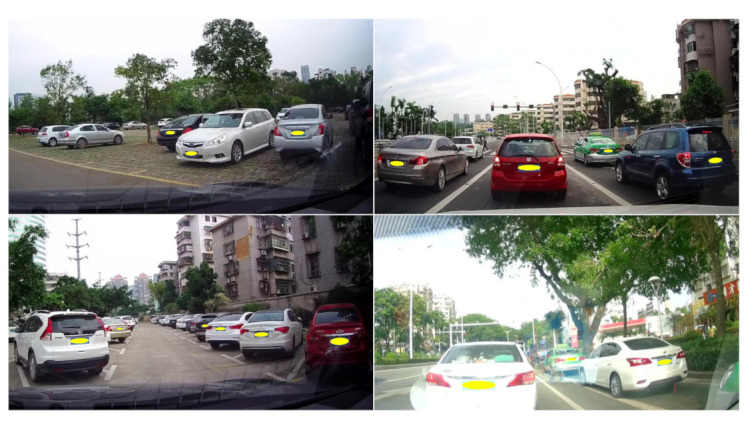
License plates with various sizes, orientations, and backgrounds in real scenarios. All the recognizable license plates are manually covered with a yellow ellipsoid to protect privacy.

License plate detection (LPD) plays an essential role in many practical applications, including electronic toll collection, traffic surveillance, and enforcement. When the image acquisition conditions (shooting distance and angle) are restricted, such as the parking toll, the LPD task is almost completely solved. However, if the image is captured in the wild, it remains challenging due to various sizes, orientations, and backgrounds. Figure 1 illustrates some license plate (LP) examples in real scenarios.

Recent LPD methods can be roughly divided into direct and indirect ways. Direct methods directly localize the license plate in the input image with handcrafted features [1,2,3,4], deep-learning features [5,6,7,8,9], or license plate recognition system [10,11]. However, detecting small-sized license plates is challenging since they only occupy a relatively small area in the whole image. Indirect methods detect the license plate using the vehicle’s proposal [12,13,14,15,16,17] or the vehicle head region [18]. The vehicle head region is manually defined as the smallest region enclosing the headlights and tires. The indirect methods can reduce the detection area and background noises, which is favorable to small-sized license plate detection. However, when the vehicle fails to be detected due to severe occlusion or nonuniform illumination, it will fail to localize the license plate.

To overcome these problems, we propose a novel network composed of an indirect branch and a direct branch. The indirect detection branch can approximately localize the license plate based on the spatial relationships between the license plate and the vehicle. Then it can refine the license plate in the local region. This way, it can significantly reduce the detection area and mitigate the adverse effects of the background noises, which is favorable to small-sized license plate detection. The direct detection branch can reduce false negatives in the indirect detection branch due to the miss of vehicles’ detection. We combine the indirect and direct branches to construct an end-to-end trainable network for license plate detection. The detection results of two branches are merged by post-processing operations, such as non-maximum suppression (NMS). Extensive experiments show that our method outperforms both the direct approach and the indirect approach.

Moreover, many methods [6,9,10,11,16,18] do not consider the orientation of the license plate, which is only applicable to specific scenarios, such as parking charges and vehicle access/exit management. When it comes to more complex scenarios, such as road scenes, if we regard the tilted license plate as the horizontal direction, it may cause errors in the subsequent license plate recognition [19,20,21,22]. Although Dong et al. [14,23] propose to detect the multidirectional license plate, these methods are very complicated due to adopting multiple separate models.

We propose to detect the multidirectional license plate by localizing the four corners of the license plate to reduce complexity. It can be easily implemented by integrating the corner prediction module into the two branches mentioned above, with no extra models. In this way, the whole detection network is still in an end-to-end trainable manner, as shown in our open-source codes [24].

Our main contributions can be summarized as:

- We propose a novel network that combines indirect and direct branches for license plate detection in the wild. The indirect detection branch utilizes vehicle–plate relation and can precisely locate the license plate in a coarse-to-fine scheme. The direct detection branch localizes the license plate in the input image directly, reducing false negatives in the indirect detection branch due to the miss of vehicles’ detection.
- We propose to detect the multidirectional license plate by localizing the four corners of the license plate. This universal detection module can be easily integrated into standard detection networks.
- Notably, the whole model is constructed in an end-to-end trainable manner. By utilizing the post-processing operations, such as NMS, the final detection results are obtained by merging the indirect and direct branches. Hence, the whole model benefits from joint learning of all tasks. To our knowledge, our model is the first one that combines indirect and direct methods into an end-to-end network for license plate detection.

The rest of this paper is organized as follows. Related work is described in Section 2. In Section 3, we describe our method in detail. Section 4 presents comparative experiments and analyses. A short discussion is presented in Section 5. The final remarks are presented in Section 6.

## 2. Related Work

### 2.1. Direct License Plate Detection

The following methods propose to detect the license plate in the input image directly. Jun et al. [25] present a morphology-based method for LPD by extracting contrast features. To solve illumination variation and background interference, Tian et al. [1] propose an Adaboost algorithm combined with a color differential model, which can detect the license plate in a coarse-to-fine manner. The literature [4,26] propose to use the edge and texture features for license plate detection. Zhou et al. [2] propose to localize the license plate by principal visual word, discovery, and local feature matching. Li et al. [3] propose a component-based method for license plate detection. This method detects candidate characters first, then constructs the spatial relationships of characters using conditional random field (CRF), and finally estimates the whole license plate. Yuan et al. [6] apply dense filters to extract all the possible candidate LP regions and then preserve true positive LPs using a cascaded classifier. Rabiah et al. [7] propose a YOLO-inspired adaptive solution with optimized parameters to enhance LPD performance. In literature [8], the license plate features from the bottom and high levels of the CNN network are extracted and integrated to achieve precise and real-time detection. Chen et al. [9] propose to detect the license plate in a separate branch to avoid the suppression effects caused by the vehicle. Xu et al. [10] use multi-level CNN features to detect multi-scale license plates. Li et al. [11] utilize Faster R-CNN [27] to detect the license plate, where the scales and shapes of anchors are designed to fit the license plate. However, these approaches are prone to fail small-sized license plates because the license plate only occupies a relatively small input image area.

### 2.2. Indirect License Plate Detection

The following methods propose to detect the license plate via vehicle–plate relation. In this way, it enables the model to focus on the potential location of the license plate and reduce disturbing background noises, which can improve the detection performance of small-sized license plates. Kim et al. [12] use R-CNN [28] to detect the vehicles first, then localize the license plate inside each vehicle. Fu et al. [13] apply the region proposal network (RPN) [27] to generate candidate vehicle proposals and then detect the license plate based on the convolutional features of the vehicle. The literature [14,15] propose a two-stage YOLOv2 [29] method for accurate license plate detection. The first stage detects the vehicle and the second stage detects the license plate in the detected vehicle region. Rayson et al. [16] utilize YOLOv2 [29] to detect all the possible vehicles and then localize all the license plates in the vehicle patches simultaneously. Sergio et al. [18] propose to detect the vehicle firstly, then detect the vehicle head region in the vehicle, and localize the license plate in each vehicle head region finally. Chen et al. [17] propose estimating the approximate location of the license plate based on the offset between the center of the license plate and the vehicle, then refine the quadrilateral bounding box of the license plate in the local region. However, these methods will inevitably fail to localize the license plate if the vehicle fails to be detected. Our method combines the advantages of both indirect LPD approaches and direct LPD approaches, where it can detect small-sized license plates via vehicle–plate relation and reduce false-negative license plates caused by wrongly detected vehicles.

### 2.3. Multidirectional License Plate Detection

Xie et al. [30] propose to predict the rotation angle for multidirectional license plate detection based on modified YOLO [31]. Han et al. [5] propose to detect the license plate with a parallelogram by predicting three corners of the license plate. Tian et al. [32] adopt a semantic segmentation network for candidate license plate extraction and then refine the oriented bounding box of the license plate. All of the methods above regard the oblique license plate as a parallelogram. However, in real scenarios, a highly oblique license plate is an arbitrary quadrangle due to perspective transformation. Dong et al. [23] present to extract license plate candidates with RPN [27] and then use R-CNN [28] to localize the four corners of the license plate. The literature [14,33] employ spatial transformer networks (STN) [34] to obtain the affine transformation parameters of the license plate and transform the oblique license plate into a horizontal direction. However, the literature [14,23] are complicated due to adopting several separate models; moreover, they demand large-scale training data for STN. Our method can localize the quadrilateral bounding box of the license plate in an end-to-end manner, with no need for large-scale training data.

## 3. Materials and Methods

We propose a novel network for license plate detection, which can effectively detect the small-sized license plate and accurately localize the multidirectional license plate in real applications. The overall architecture is described in Section 3.1. The indirect detection branch can precisely detect the small-sized license plate, as described in Section 3.2. The direct detection branch can reduce the false-negative license plate in the indirect detection branch due to incorrectly detected vehicles, as described in Section 3.3. The whole network is constructed in an end-to-end trainable manner, as described in Section 3.4. The detection results of these two detection branches are merged by post-processing operations, such as NMS, as described in Section 3.5.

### 3.1. Overall Architecture

**Figure 2:**
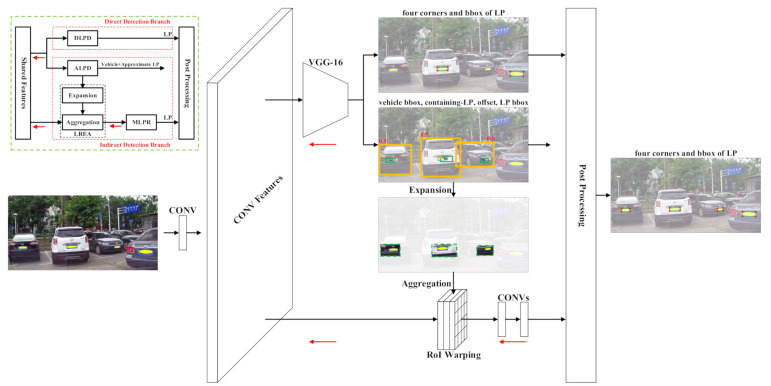
A thumbnail of the overall architecture is shown in the top-left corner (DLPD: Direct License Plate Detection; ALPD: Approximate License Plate Detection; LREA: Local Region Estimation and Aggregation; MLPR: Multidirectional License Plate Refinement). All the recognizable license plates are manually covered with a yellow ellipsoid to protect privacy.

The overall architecture is illustrated in Figure 2. The network is constructed with two detection branches, i.e., indirect detection branch and direct detection branch. In the indirect detection branch, the approximate location and size of the license plate are predicted at the ALPD stage, where the center of the license plate (green circle) is obtained based on the offset (purple arrow) between the center of the license plate and the vehicle (orange circle). Moreover, the probability of the vehicle containing a license plate (red number) is predicted simultaneously. At the LREA stage, the local region of LP is obtained by expanding the LP region, and all the expanded LP regions (green dashed rectangle) are resized and aggregated into feature patches via differentiable region of interest (RoI) warping [35] for batch operation. At the MLPR stage, the quadrilateral (red circle) and horizontal (green rectangle) bounding boxes of the license plate are detected simultaneously in the local region of LP. In the direct detection branch, the license plate is directly detected in the input image at the DLPD stage. The DLPD and ALPD modules share the same backbone network but different detection head networks. Finally, the detection results of two branches are merged by post-processing operations, such as NMS. The network can be trained in an end-to-end manner, where the red arrows denote the backpropagation gradients.

### 3.2. Indirect Detection Branch

The indirect detection branch predicts the approximate location of the license plate utilizing spatial vehicle–plate relationships firstly (Section 3.2.1), then estimates the local region by expanding the LP region followed by an aggregation operation (Section 3.2.2), and refines the quadrilateral and horizontal bounding boxes of the license plate in the local region finally (Section 3.2.3). This multi-level design enables the model to focus on the potential location of the license plate and reduce the disturbing background noises.

#### 3.2.1. Approximate License Plate Detection (ALPD)

At this stage, the approximate location of the license plate is estimated according to the vehicle–plate relation. At first, the vehicle is detected, so the center of the vehicle is determined. After that, the location of the license plate is obtained based on the offset between the center of the license plate and the vehicle. Meanwhile, the size of the license plate is directly predicted in the input image. According to the center and size, the license plate is approximately detected. In addition, the probability of the vehicle containing a license plate is predicted simultaneously. As shown in Figure 2, the location and size of the license plate are not accurate in general cases because the license plate only occupies a relatively small area in the large input image.

The ALPD module is based on SSD [36] for multi-task learning, which is the same as SSD512 [36] except for the training objective. The training objective of the ALPD module is defined as Equation (1), including five losses: vehicle classification loss $L_{cls}\left(c\right)$, vehicle regression loss $L_{reg}\left(p,g\right)$, offset loss $L_{off}\left(p,g\right)$, LP size loss $L_{size}\left(p,g\right)$, and containing-LP loss $L_{con\_lp}\left(p,g\right)$.

(1) $$L_{1}\left(c,p,g\right)=\frac{1}{N_{v}}\left[L_{cls}\left(c\right)+L_{reg}\left(p,g\right)+L_{off}\left(p,g\right)+L_{size}\left(p,g\right)+L_{con\_lp}\left(p,g\right)\right],$$

where $N_{v}$ is the number of matched anchor boxes with the ground-truth vehicles, *c* is the vehicle presence confidence, *p* is the predicted parameters, and *g* is the ground-truth parameters.

The training objective of vehicle detection is derived from SSD [36], including classification loss (i.e., Equation (2)) and regression loss (i.e., Equation (3)). The classification loss is the softmax loss over categories ζ∈{vehicle,background}. The regression loss is the smooth L1 loss [37] of the foreground category $\zeta^{+}=vehicle$, which regresses to offsets for the center (cx,cy), width (w), and height (h) of the matched anchor box.

(2) $$L_{cls}\left(c\right)=-\sum_{i=1}^{N_{v}}\sum_{\zeta }\log\left(c_{i}^{\zeta }\right)\;\;\;\;\;c_{i}^{\zeta }=\frac{exp\left({\hat{c_{i}}}^{\zeta }\right)}{\sum_{\zeta }exp\left({\hat{c_{i}}}^{\zeta }\right)},$$

(3) $$L_{reg}\left(p,g\right)=\sum_{i=1}^{N_{v}}\sum_{m\in \left\{cx,cy,w,h\right\}}I_{ij}^{\zeta^{+}}Smooth_{L1}\left(p_{i}^{m}-g_{j}^{m}\right),$$

where $I_{ij}^{\zeta^{+}}\in \left\{0,1\right\}$ is the indicator of whether the *i*th anchor box matches the *j*th ground-truth box.

The offset and size losses are the smooth L1 loss between the predicted parameters (p) and the ground-truth parameters (g) based on the matched anchor boxes, as shown in Equations (4) and (5). The vehicle must contain a license plate; otherwise, the losses $L_{off}\left(p,g\right)$ and $L_{size}\left(p,g\right)$ are 0 by setting $g_{j}^{+}=0$. This way, it can avoid learning false-positive predictions during training.

(4) $$L_{off}\left(p,g\right)=\sum_{i=1}^{N_{v}}\sum_{m\in \left\{off_{x},off_{y}\right\}}I_{ij}^{\zeta^{+}}g_{j}^{+}Smooth_{L1}\left(p_{i}^{m}-g_{j}^{m}\right),$$

(5) $$L_{size}\left(p,g\right)=\sum_{i=1}^{N_{v}}\sum_{m\in \left\{lp_{w},lp_{h}\right\}}I_{ij}^{\zeta^{+}}g_{j}^{+}Smooth_{L1}\left(p_{i}^{m}-g_{j}^{m}\right),$$

where $off_{x}$ and $off_{y}$ are the offsets between the center of the license plate and the vehicle in x-direction and y-direction, $lp_{w}$ and $lp_{h}$ are the width and height of the license plate, and $g_{j}^{+}\in \left\{0,1\right\}$ is the indicator of whether the *j*th vehicle contains a license plate.

Moreover, the probability of the vehicle containing a license plate can be used to reduce false positives of the license plate. A license plate will be detected only when the probability is greater than a certain threshold, and the threshold is empirically set to 0.5. During training, the vehicles with very small-sized or invisible license plates (occlusion, far shooting-distance, etc.) are regarded as without license plates; otherwise, the vehicles are considered as containing a license plate. The containing-LP probability is optimized by the binary cross-entropy loss (i.e., Equation (6)).

(6) $$L_{con\_lp}\left(p,g\right)=-\sum_{i=1}^{N_{v}}\left[g_{j}^{+}\cdot \log\left(\sigma \left(p_{i}^{+}\right)\right)+\left(1-g_{j}^{+}\right)\cdot \log\left(1-\sigma \left(p_{i}^{+}\right)\right)\right],$$

where σ is a sigmoid function to limit the predicted containing-LP probability $p_{i}^{+}\in \left[0,1\right]$ in case of loss divergence.

#### 3.2.2. Local Region Estimation and Aggregation (LREA)

After the ALPD stage, there is a large deviation between the predicted license plate and the ground truth. We should make a further refinement to get more precise detection results, i.e., fine-tuning the license plate in the local region around the license plate. Based on the center and size of the license plate, we obtain the local region by merely expanding the license plate region with a preset ratio, enclosing the license plate and little background. The license plate occupies a relatively larger area in the local region than in the input image so that the subsequent refinement network can get more precise detection results.

There are many license plate regions obtained from different vehicles simultaneously. All the region features are extracted from the first convolutional layer, ensuring the whole network is constructed in an end-to-end manner. The first convolutional layer preserves the same size as the input image, which retains sufficient spatial information to detect small-sized license plates. Furthermore, all the LP regions are resized and aggregated via differentiable RoI warping [35] for batch operation, ensuring all the license plates are detected simultaneously to reduce the running time.

#### 3.2.3. Multidirectional License Plate Refinement (MLPR)

**Figure 3:**
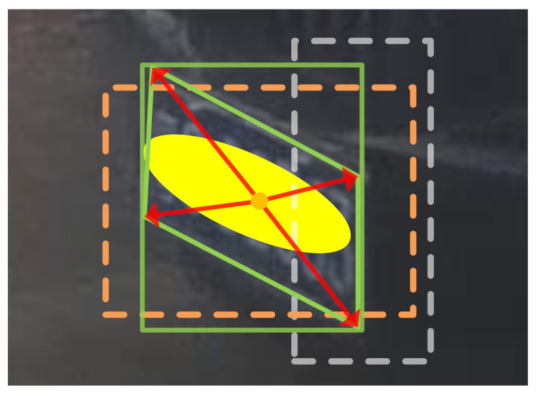
The four corners of the license plate are regressed based on the offsets (red arrow) from the center (orange circle) of the matched anchor box (dashed orange rectangle). The matched anchor box is determined by the intersection over union (IoU) with the horizontal ground-truth box (solid green rectangle). The negative anchor box (dashed gray rectangle) is neglected due to low IoU. The license plate is manually covered with a yellow ellipsoid to protect privacy.

In the local region, the quadrilateral and horizontal bounding boxes of the license plate are detected simultaneously. The quadrilateral bounding box is obtained by regressing the four corners of the license plate based on the matched anchor box, as illustrated in Figure 3. The matched anchor box is determined by the intersection over union (IoU) with the horizontal ground-truth box. The horizontal bounding box is used for NMS because of the fast computing speed. Compared with the ALPD module, the detection results of the MLPR module are more accurate.

The MLPR module has only 6 convolutional layers because the LPD task in the local region is relatively simple. Please refer to our open-source codes [24] for more details. The training objective of the MLPR module is defined as Equation (7), including three parts: LP classification loss $L_{cls}\left(c^{\prime }\right)$, LP regression loss $L_{reg}\left(p^{\prime },g^{\prime }\right)$, and LP corner loss $L_{corner}\left(p^{\prime },g^{\prime }\right)$.

(7) $$L_{2}\left(c^{\prime },p^{\prime },g^{\prime }\right)=\frac{1}{N_{lp}^{\prime }}\left[L_{cls}\left(c^{\prime }\right)+L_{reg}\left(p^{\prime },g^{\prime }\right)+L_{corner}\left(p^{\prime },g^{\prime }\right)\right],$$

where $N_{lp}^{\prime }$ is the number of matched anchor boxes with the horizontal LP ground-truth boxes, $c^{\prime }$ is the LP presence confidence, $p^{\prime }$ is the predicted LP parameter, and $g^{\prime }$ is the LP ground-truth parameter.

The losses of the horizontal bounding box $L_{cls}\left(c^{\prime }\right)$ and $L_{reg}\left(p^{\prime },g^{\prime }\right)$ are the same as vehicle detection except for the foreground category being LP, as shown in Equation (2) and Equation (3). As shown in Equation (8), the corner loss of the quadrilateral bounding box is the smooth L1 loss of the foreground category ${\zeta^{\prime }}^{+}=license\;plate$, which regresses to offsets between the center of the matched anchor box and the four corners of the license plate.

(8) $$L_{corner}\left(p^{\prime },g^{\prime }\right)=\sum_{i=1}^{N_{lp}^{\prime }}\sum_{m\in \left\{tl_{x},tl_{y},tr_{x},tr_{y},br_{x},br_{y},bl_{x},bl_{y}\right\}}I_{ij}^{{\zeta^{\prime }}^{+}}Smooth_{L1}\left({p^{\prime }}_{i}^{m}-{g^{\prime }}_{j}^{m}\right),$$

where $m\in \left\{tl_{x},tl_{y},tr_{x},tr_{y},br_{x},br_{y},bl_{x},bl_{y}\right\}$ are the four corners of the license plate, i.e., top-left, top-right, bottom-right, and bottom-left.

### 3.3. Direct Detection Branch

Direct License Plate Detection (DLPD)

The DLPD module can directly detect the license plate in the input image. In this way, small-sized license plates can not always be detected. However, in some cases, when the license plate fails to be detected in the indirect detection branch due to incorrectly detected vehicles, the DLPD module can reduce the false-negative license plate. The DLPD module is similar to the MLPR module described in Section 3.2.3. One significant difference is that the license plate is directly detected in the input image, not in the local region of the license plate. In addition, the backbone network of the DLPD module is the same as SSD [36] with 25 convolutional layers; the backbone network of the MLPR module only consists of 6 convolutional layers, as described in Section 3.2.3.

According to [9], it is difficult to effectively detect the vehicle and license plate simultaneously due to their subordinate relationships. This issue is caused by feature interaction between the vehicle and license plate in the traditional anchor-based detection method, such as SSD [36]. To solve this problem, we construct two separate detection branches for the DLPD and ALPD modules, respectively, as shown in Figure 2. These two modules share the same backbone network (i.e., VGG-16 [38] and extra layers) but different head networks. Please refer to our open-source codes [24] for more details. In this way, we can eliminate the adverse effects on the license plate caused by the vehicle.

Similar to the MLPR module, the training objective of the DLPD module is defined as Equation (9), including LP classification loss $L_{cls}\left(c^{\prime \prime }\right)$, LP regression loss $L_{reg}\left(p^{\prime \prime },g^{\prime \prime }\right)$, and LP corner loss $L_{corner}\left(p^{\prime \prime },g^{\prime \prime }\right)$.

(9) $$L_{3}\left(c^{\prime \prime },p^{\prime \prime },g^{\prime \prime }\right)=\frac{1}{N_{lp}^{\prime \prime }}\left[L_{cls}\left(c^{\prime \prime }\right)+L_{reg}\left(p^{\prime \prime },g^{\prime \prime }\right)+L_{corner}\left(p^{\prime \prime },g^{\prime \prime }\right)\right].$$

### 3.4. End-to-End Trainable Detection Network

By integrating the indirect and direct detection branches, we develop an end-to-end trainable network for license plate detection, which can effectively detect the small-sized license plate and accurately localize the multidirectional license plate in real applications. Combining Equations (1), (7), and (9), the loss of the whole network is shown in Equation (10), where α and β are simply set to 1 to balance these loss terms.

(10) $$L=L_{1}\left(c,p,g\right)+\alpha L_{2}\left(c^{\prime },p^{\prime },g^{\prime }\right)+\beta L_{3}\left(c^{\prime \prime },p^{\prime \prime },g^{\prime \prime }\right).$$

**Figure 4:**
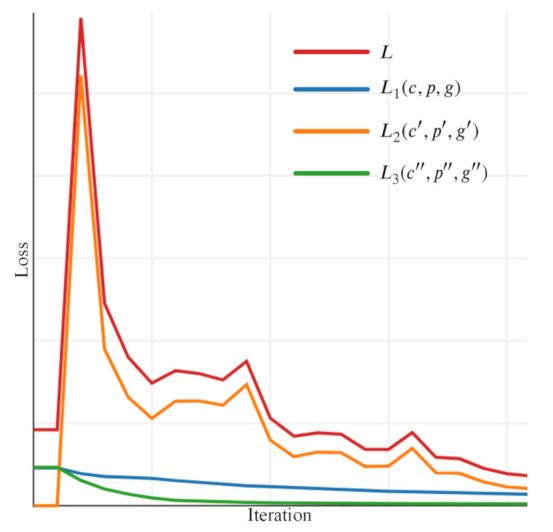
Training loss. $L_{1}\left(c,p,g\right)$ is the loss of the ALPD module in the indirect detection branch. $L_{2}\left(c^{\prime },p^{\prime },g^{\prime }\right)$ is the loss of the MLPR module in the indirect detection branch. $L_{3}\left(c^{\prime \prime },p^{\prime \prime },g^{\prime \prime }\right)$ is the loss of the DLPD module in the direct detection branch. *L* is the total loss of the end-to-end network.

Figure 4 illustrates the loss changes during training, including $L_{1}$ and $L_{2}$ of the indirect detection branch as well as $L_{3}$ of the direct detection branch. During end-to-end training, the ALPD module can be optimized to detect the vehicle and approximate location of the license plate. Meanwhile, the license plate can be directly detected in the input image by the DLPD module. After training for some iterations, the MLPR module starts to refine the location of the license plate in the local region; then, the entire network will be optimized simultaneously. Specifically, during the first few training iterations, $L_{1}$ and $L_{3}$ go down, and $L_{2}$ remains zero because the untrained ALPD module can not estimate the location of the license plate; then, $L_{2}$ goes up dramatically because the ALPD module can approximately localize the license plate, and the MLPR module starts learning to regress the four corners of the license plate in the local region; finally, the total loss *L* goes down steadily because the indirect and direct detection branches are optimized simultaneously.

### 3.5. Post Processing

**Figure 5:**
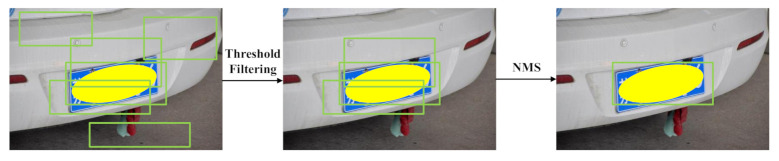
Post-processing operations. The green rectangles are the possible detection results predicted by the network. After threshold filtering and non-maximum suppression (NMS), we can get the final detection results. The license plate is manually covered with a yellow ellipsoid to protect privacy.

Figure 5 illustrates the post-processing operations. We can filter the most useless detection results by thresholding the confidence predicted by the network. After threshold filtering, the post-processing module can merge the detection results from two detection branches via NMS, removing duplicate detections. Instead of the quadrilateral bounding box, the horizontal bounding box of the license plate is used for NMS because of its faster computing speed. As shown in Section 4.7, the final detection results are mainly from the indirect detection branch because of its ability to detect small-sized license plates. In some cases, the direct detection branch can reduce the false-negative license plate in the indirect detection branch due to incorrectly detected vehicles. In this way, the network can detect the license plate with both high Precision and Recall rates.

## 4. Results

The backbone network of the DLPD and ALPD modules follows SSD512 [36], which is initialized with the ILSVRC CLS-LOC dataset [39]. The backbone network of the MLPR module is initialized with the Xavier initializer [40]. Following SSD [36], we adopt the data augmentation and hard negative mining strategies for model robustness. We train the model for 60 K iterations using Adam [41] with initial learning rate $10^{-4}$, 0.9 $\beta_{1}$ momentum, 0.99 $\beta_{2}$ momentum, $5\times 10^{-4}$ weight decay, and batch size 32. The learning rate is decreased by 10 times at the 20K and 40K iterations.

### 4.1. Datasets

TILT720. We use a driving recorder to capture road videos with a resolution of 720×1280, including the scenes of residential areas, highways, and expressways. After keyframe extraction and deduplication, we get 1033 valid images. We carefully annotate all the visible vehicles and license plates, including their subordinate relationships. The vehicle is annotated with the top-left and bottom-right points, forming a horizontal bounding box. The license plate is annotated with the four corners, forming a quadrilateral bounding box. The horizontal bounding box of the license plate is the minimal horizontal bounding rectangle of the quadrilateral bounding box. For simplicity, we name this dataset TILT720 (mulTidirectional lIcense pLate deTection dataset 720P). All the images are randomly divided into the training-validation set and test set in the proportion of 9:1.

TILT1080. Similar to the TILT720, we obtain the TILT1080 with another driving recorder. The TILT1080 contains 4112 images, and all the images have a size of 1080×1920. All the images are randomly divided into the training-validation set and test set in the proportion of 9:1.

### 4.2. Evaluation Protocols

**Figure 6:**
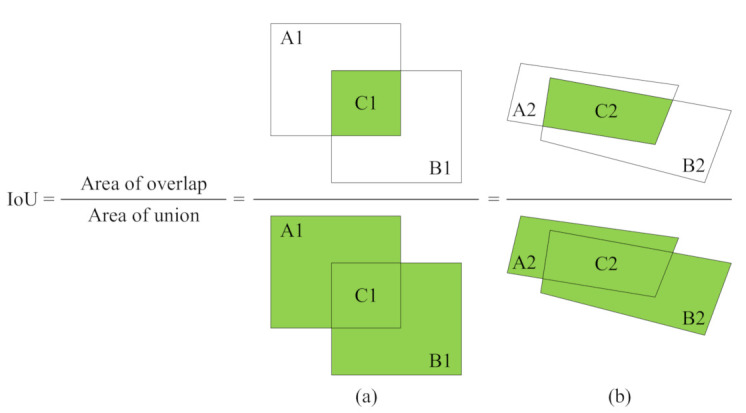
(**a**) IoU between two horizontal boxes. (**b**) IoU between two quadrilateral boxes.

We adopt the Average Precision (AP) to evaluate the horizontal bounding box. Specifically, we use the 11-points computation of VOC2007 [42] with different IoU thresholds (i.e., 0.5 and 0.75). As shown in Figure 6a, the IoU is calculated between two horizontal boxes, i.e., IoU = $\frac{C1}{A1+B1-C1}$.

Moreover, we adopt the Precision, Recall, and $F_{1}$-score to evaluate the quadrilateral bounding box. With the confidence threshold 0.5, a quadrilateral bounding box is correct only when its IoU with the quadrilateral ground-truth box is greater than a certain threshold. As shown in Figure 6b, the IoU is calculated between two quadrilateral boxes, i.e., IoU = $\frac{C2}{A2+B2-C2}$.

### 4.3. Ablation Study

As shown in Table 1, we adopt the ALPD module as the benchmark model. The ALPD module is described in Section 3.2.1, which is the first step of the indirect detection branch and can approximately estimate the license plate in the input image. The module only achieves very low AP on all the test sets, especially for the IoU threshold 0.75. After only adding the MLPR module, the detection performance worsens because the license plate is refined in the region that cannot completely enclose the license plate. According to [17], we further add the LREA module, where the license plate region is expanded to 3 times. In this way, the license plate can be refined in the local region that can completely enclose the license plate with a little background. The ALPD, LREA, and MLPR modules assemble the indirect detection branch, improving the AP by 10%–20% with different IoU thresholds compared with the ALPD module.

The DLPD module can directly detect the license plate in the input image, which achieves comparable performance with the indirect detection branch with a small IoU threshold; however, with a large IoU threshold, the performance is much lower. The DLPD module cannot accurately localize the license plate in the large input image because of more background noises, making it difficult to detect the small-sized license plate.

Combining the indirect and direct detection branches, we get the whole detection network, which achieves higher AP on all the test sets with different IoU thresholds. The network can detect the small-sized license plate via vehicle–plate relation and reduce the false-negative license plate caused by incorrectly detected vehicles.

Moreover, the ALPD module, the indirect detection branch, and the whole detection network have almost the same vehicle detection performance as the vanilla SSD [36]. This way, it proves our method can continuously improve the license plate detection performance while maintaining the vehicle detection performance [43,44,45,46,47].

### 4.4. Evaluation of Horizontal Bounding Box

**Figure 7:**
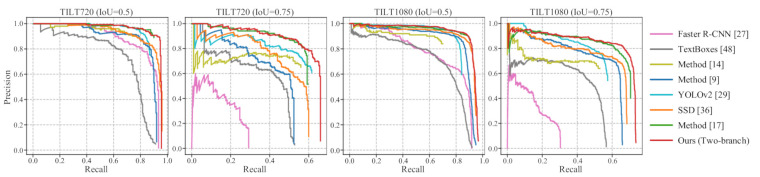
The Precision–Recall curve of different methods. The title of each graph indicates the dataset and IoU threshold for testing. Our method achieves the best performance for all the test sets and IoU thresholds in terms of the area under the curve (AUC). Moreover, our method achieves the best Recall rate, according to the Recall-axis.

We do not consider the orientation of the license plate and calculate the AP based on the detected horizontal bounding box in this subsection. We compare [9,14,17,27,29,36,48] with our proposed method. The backbone network and input size of Faster R-CNN [27], SSD [36], and the method in [17] are the same as our method, while the settings of methods [9,14,29,48] remain unchanged. Except for [14] (The authors released models for license plate detection at https://github.com/sergiomsilva/alpr-unconstrained (accessed on 3 February 2021).), all other methods are trained with the trainval set of TILT720 and TILT1080, respectively. As shown in Table 2, our method achieves the best performance for all the test sets and IoU thresholds. Moreover, as shown in Figure 7, our method has the best performance considering the area under the curve (AUC) and achieves the highest Recall rate according to the Recall-axis. SSD [36] can directly detect the license plate in the input image and achieve comparable performance with our method with a small IoU threshold. However, with a large IoU threshold, SSD [36] significantly lags because the background noises from the large input image can disturb the detection of the license plate. The method [17] can significantly improve the AP by detecting the license plate in the local region around the license plate, which can greatly reduce the background noises; nevertheless, it will inevitably fail the license plate if the vehicle fails to be correctly detected. Our method combines the advantages of method [17] and SSD [36] by integrating two detection branches, i.e., indirect branch and direct branch. Same as [17], the indirect detection branch can detect the license plate in the local region. Furthermore, the direct detection branch can reduce the false-negative license plate in the indirect detection branch due to incorrectly detected vehicles. In this way, our method achieves higher Precision and Recall rates compared with the method in [17] and SSD [36].

### 4.5. Evaluation of Multidirectional License Plate

We calculate the Precision, Recall, and $F_{1}$-score based on the predicted quadrilateral bounding box. For the methods [9,27,29,36,48] that can only detect the horizontal bounding box, we only compare the best SSD [36] with our proposed method. As shown in Table 3, our method achieves the best $F_{1}$-score for all the test sets with different IoU thresholds. SSD [36] achieves relatively poor performance, because the detection results of SSD [36] have very low IoU with the quadrilateral ground-truth box. Furthermore, like the DLPD module, we upgrade SSD [36] and make it capable of directly detecting the four corners of the license plate in the input image (SSD+FC). SSD+FC can achieve much better performance than the vanilla SSD [36], especially for the large IoU threshold. However, SSD+FC suffers low Recall because of the background noises. As shown in Section 4.4, our method combines the advantages of method [17] and SSD+FC, and can precisely detect the multidirectional license plate with a higher Recall rate.

### 4.6. Evaluation of Small-Sized License Plate

**Figure 8:**
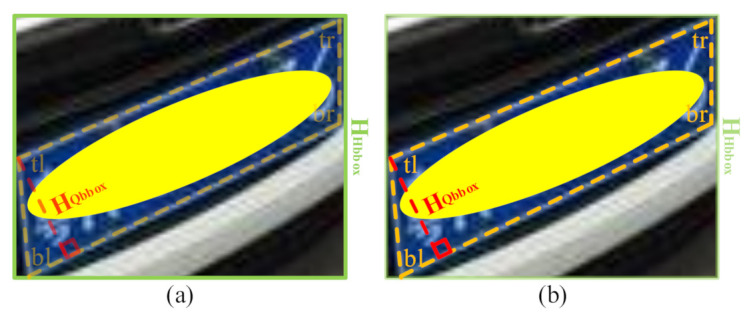
(**a**) The height of the horizontal bounding box $H_{Hbbox}$. (**b**) The distance between the top-left corner and the straight line formed by the bottom-left and bottom-right corners $H_{Qbbox}$. In this work, the height of the license plate is defined as $H_{Qbbox}$. The license plate is manually covered with a yellow ellipsoid to protect privacy.

According to the height of the license plate, we divide the test set into three parts, i.e., small, medium, and large. To avoid large deviation, we define the height of the multidirectional license plate as $H_{Qbbox}$ in Figure 8b. As for our datasets, we define the small LP with height∈(0,16] pixels, the medium LP with height∈(16,32] pixels, and the large LP with height∈(32,+∞) pixels.

We use the Recall to evaluate the multiscale detection performance. As shown in Table 4, our method achieves the best Recall rate for almost all the sizes of different datasets with different IoU thresholds. Compared with the benchmark models SSD [36] and SSD+FC, our method achieves a large performance improvement, especially for the small- and medium-sized license plate. Same as [17], our method can effectively detect the small-sized license plate in the local region, which greatly improves the Recall rate. Furthermore, the direct detection branch can reduce the false-negative license plate due to incorrectly detected vehicles, which further improves the Recall rate based on method [17]. However, the post-processing module may remove true-positive predictions (i.e., 30.53 vs. 29.47 of TILT720), and this is what we should improve in future work.

### 4.7. Qualitative Results

**Figure 9:**
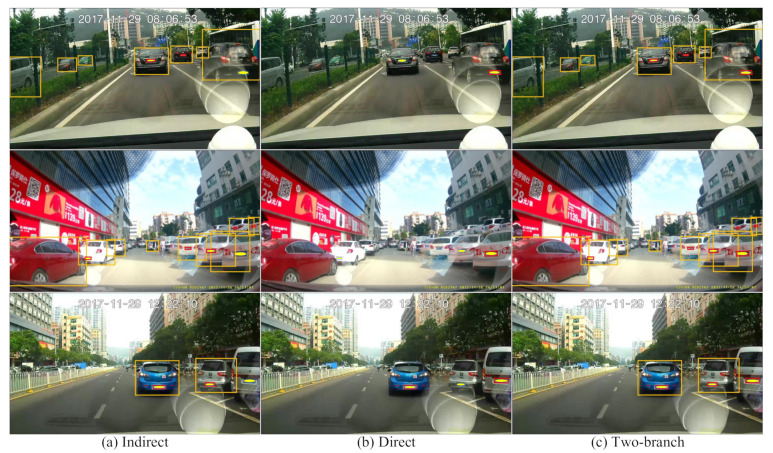
Detection results. The orange rectangle represents the horizontal bounding box of the vehicle. The red quadrangle indicates the quadrilateral bounding box of the license plate. Our method merges the detection results of the indirect and direct detection branches. All the recognizable license plates are manually covered with a yellow ellipsoid to protect privacy.

Some qualitative detection results are illustrated in Figure 9. The license plate can be detected via vehicle–plate relation in the indirect detection branch, especially for the small-sized license plate. However, when many vehicles are close to each other, some vehicles may be detected with a large deviation, as shown in the first two images. In addition, in some cases, the vehicle fails to be detected due to boundary truncation, as shown in the third image. In these cases, the license plate cannot be detected in the indirect detection branch. Meanwhile, the license plate can be directly detected in the input image in the direct detection branch. However, due to the disturbing background noises, the direct detection branch can only detect relatively large and horizontal license plate.

By combing these two detection branches with post-processing operations, such as NMS, we get the final detection results. As can be seen, these two detection branches are complementary to each other. The indirect detection branch can detect most of the license plates; in some cases, the direct detection branch can reduce the false-negative license plate in the indirect detection branch due to undetected vehicles or vehicles with large deviations.

## 5. Discussions

In summary, we have verified the effectiveness of our proposed method to detect multiscale and multidirectional license plates. The indirect detection branch can detect most license plates via vehicle–plate relation. The direct detection branch can reduce false-negative license plates when the vehicle is wrongly detected in the indirect detection branch. Both detection branches can detect multidirectional license plates by regressing the four corners of the license plate. After tilt correction, we can improve the license plate recognition performance [19,20,21,22]. The license plate information can be applied to barrier access control [20,22], vehicle target detection [49], vehicle re-identification [50], etc. Moreover, the location of the license plate can be used for vehicle trajectory prediction [51] via license plate detection and tracking.

**Figure 10:**
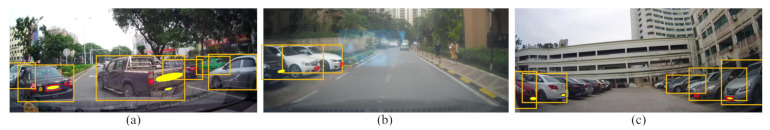
Failed examples. The orange rectangle represents the horizontal bounding box of the vehicle. The red quadrangle indicates the quadrilateral bounding box of the license plate. (**a**) Failed due to various illuminations. (**b**) Failed due to vehicle occlusion or boundary truncation. (**c**) Failed due to various orientations. All the recognizable license plates are manually covered with a yellow ellipsoid to protect privacy.

However, in some cases, the proposed method fails to detect the license plate, and Figure 10 illustrates some failed examples. In these cases, both the indirect and direct detection branches fail to detect the license plate, especially the indirect detect branch. As shown in Figure 10a, the license plate of the middle pick-up truck is undetected due to various illuminations caused by the mirror reflection on the front windshield, which may be caused by never seeing such images during training. As shown in Figure 10b, the license plate of the leftmost black vehicle is undetected due to the miss of vehicles’ detection. The two close vehicles are detected with only one box due to vehicle occlusion or boundary truncation. We will improve the vehicle detection performance to enhance the LPD performance in future work. As shown in Figure 10c, the left two license plates are undetected due to various orientations, which may be caused by a large deviation of the center offset between the vehicle and license plate. In this case, the approximate location of the license plate is wrongly estimated, so the next refinement stage cannot localize the license plate.

## 6. Conclusions

We propose an end-to-end trainable network for license plate detection, which can effectively detect the small-sized license plate and accurately localize the multidirectional license plate in real applications. The network is composed of two detection branches, i.e., indirect branch and direct branch. The indirect detection branch can detect the license plate via vehicle–plate relation in a coarse-to-fine scheme. The direct detection branch can directly detect the license plate in the input image. All these branches can detect multidirectional license plates by regressing the four corners of the license plate. The final detection results are obtained by merging these two detection branches via post-processing operations, such as NMS. To our knowledge, our proposed method is the first one that combines indirect and direct methods into an end-to-end network for license plate detection. Experiments show that the indirect detection branch can detect most license plates, especially the small-sized license plate. The direct detection branch can reduce the false-negative license plate in the indirect detection branch due to incorrectly detected vehicles. In this way, our proposed method achieves both high Precision and Recall rates.

## Figures and Tables

**Table 1:** Ablation study of different datasets with different IoU thresholds. The values represent the Average Precision (AP) based on the horizontal bounding box.

Method	LREA	MLPR	DLPD	IoU = 0.5		IoU = 0.75	
				TILT720	TILT1080	TILT720	TILT1080
ALPD				76.71%	77.71%	26.27%	35.27%
Indirect		*√*		40.35%	40.62%	7.48%	10.76%
	*√*	*√*		89.19%	87.67%	54.51%	56.92%
Direct			*√*	86.85%	86.01%	47.52%	53.34%
Two-branch	*√*	*√*	*√*	89.30%	87.79%	56.54%	57.94%

**Table 2:** Comparative experiments of the horizontal bounding box. The values represent the AP based on the horizontal bounding box.

Method	IoU = 0.5		IoU = 0.75	
	TILT720	TILT1080	TILT720	TILT1080
Faster R-CNN [27]	81.65%	73.88%	13.63%	14.29%
TextBoxes [48]	69.67%	67.56%	37.24%	38.66%
Method [14]	74.67%	64.78%	42.67%	38.61%
Method [9]	84.05%	82.05%	45.35%	53.42%
YOLOv2 [29]	80.80%	79.58%	51.66%	49.32%
SSD [36]	86.63%	86.34%	47.06%	53.88%
Method [17]	89.19%	87.67%	54.51%	56.92%
Ours (Direct)	86.85%	86.01%	47.52%	53.34%
Ours (Indirect)	89.13%	87.11%	54.48%	56.96%
Ours (Two-branch)	89.30%	87.79%	56.54%	57.94%

**Table 3:** Comparative experiments of the multidirectional license plate. The values are calculated based on the quadrilateral bounding box.

Method	TILT720 (IoU = 0.5/0.75)			TILT1080 (IoU = 0.5/0.75)		
	Precision	Recall	$F_{1}$-Score	Precision	Recall	$F_{1}$-Score
SSD [36]	98.66/65.10	58.80/38.80	73.68/48.62	93.88/75.92	40.38/30.07	56.47/43.08
Method [14]	88.79/53.27	76.00/45.60	81.90/49.14	83.53/55.08	68.97/45.48	75.55/49.83
SSD+FC	97.47/75.32	61.60/47.60	75.49/58.33	97.57/84.67	42.61/36.98	59.32/51.48
Method [17]	90.61/60.41	88.80/59.20	89.70/59.80	88.17/61.51	87.89/61.32	88.03/61.42
Ours (Direct)	98.69/82.31	60.40/48.40	74.94/60.96	96.96/85.95	44.00/39.00	60.53/53.66
Ours (Indirect)	88.93/60.87	90.00/61.60	89.46/61.23	88.72/61.65	87.78/61.00	88.25/61.32
Ours (Two-branch)	89.68/61.90	90.40/62.40	90.04/62.15	87.85/62.09	89.16/63.02	88.50/62.55

**Table 4:** Comparative experiments of the multiscale license plate. The values represent the Recall based on the quadrilateral bounding box.

Method	TILT720 (IoU = 0.5/0.75)			TILT1080 (IoU = 0.5/0.75)		
	Large	Medium	Small	Large	Medium	Small
SSD [36]	88.46/69.23	74.42/56.59	29.47/6.32	76.43/59.24	45.28/34.45	10.87/5.43
Method [14]	92.31/88.46	85.27/62.02	58.95/11.58	88.54/82.80	78.74/54.33	39.86/7.97
SSD+FC	96.15/92.31	77.52/65.12	30.53/11.58	80.25/73.25	47.83/42.13	11.59/6.88
Method [17]	96.15/88.46	98.45/78.29	73.68/25.26	99.36/86.62	96.65/77.17	65.22/17.75
Ours (Direct)	96.15/92.31	79.07/67.44	25.26/10.53	82.17/76.43	50.20/45.47	10.87/5.80
Ours (Indirect)	96.15/88.46	98.45/79.07	76.84/30.53	99.36/84.71	96.65/77.17	64.86/17.75
Ours (Two-branch)	96.15/92.31	98.45/80.62	77.89/29.47	100.00/86.62	98.43/80.12	65.94/18.12

## Data Availability

The data presented in this study are available on request from the corresponding author. The data are not publicly available due to privacy.

## Abbreviations

The following abbreviations are used in this manuscript:

LP	License plate
LPD	License plate detection
CRF	Conditional random field
RPN	Region proposal network
STN	Spatial transformer networks
IoU	Intersection over union
RoI	Region of interest
SSD	Single shot multibox detector
YOLO	You only look once
VGG	Visual geometry group
NMS	Non-maximum suppression
DLPD	Direct license plate detection
ALPD	Approximate license plate detection
LREA	Local region estimation and aggregation
MLPR	Multidirectional license plate refinement
FC	Four corners
